# Exploring the definition of «acute» neck pain: a prospective cohort observational study comparing the outcomes of chiropractic patients with 0–2 weeks, 2–4 weeks and 4–12 weeks of symptoms

**DOI:** 10.1186/s12998-017-0154-y

**Published:** 2017-08-16

**Authors:** Luana Nyirö, Cynthia K. Peterson, B. Kim Humphreys

**Affiliations:** 1 Department of Chiropractic Medicine, Orthopaedic University Hospital Balgrist, Forchstrasse 340, 8008 Zürich, Switzerland; 2 University of Zurich, Rämistrasse 71, 8006 Zürich, Switzerland

**Keywords:** Neck pain mechanical, Treatment outcome, Chiropractic, spinal manipulative therapy, Acute

## Abstract

**Background:**

Neck pain is a common complaint in chiropractic patients. Amongst other baseline variables, numerous studies identify duration of symptoms as a strong predictor of outcome in neck pain patients. The usual time frame used for ‘acute’ onset of pain is between 0 and 4 weeks. However, the appropriateness of this time frame has been challenged for chiropractic low back pain patients. Therefore, the purpose of this study was to compare outcomes in neck pain patients with 0–2 vs 2–4 and 4–12 weeks of symptoms undergoing chiropractic treatment.

**Methods:**

This is a prospective cohort observational study with 1 year follow-up including 495 patients whose data was collected between October 2009 and March 2015. Patients were divided into high-acute (0–2 weeks), mid-acute (2–4 weeks) and subacute (4–12 weeks) corresponding to duration of their symptoms at initial treatment. Patients completed the numerical pain rating scale (NRS) and Bournemouth questionnaire for neck pain (BQN) at baseline. At follow-up time points of 1 week, 1 month, 3 months, 6 months and 1 year the NRS and BQN were completed along with the Patient Global Impression of Change (PGIC) scale. The PGIC responses were dichotomized into ‘improved’ and ‘not improved’ patients and compared between the 3 subgroups. The Chi-square test was used to compare improved patients between the 3 subgroups and the unpaired Student’s t-test was used for the NRS and BQN change scores.

**Results:**

The proportion of patients ‘improved’ was only significantly higher for patients with symptoms of 0–2 weeks compared to 2–4 weeks at the 1 week outcome time point (*p* = 0.015). The NRS changes scores were significantly greater for patients with 2–4 weeks of symptoms compared to 4–12 weeks of symptoms only at 1 week (*p* = 0.035).

**Conclusions:**

The time period of 0–4 weeks of symptoms as the definition of “acute” neck pain should be maintained. Independent of the exact duration of symptoms, medium-term and long-term outcome is favourable for acute as well as subacute neck pain patients.

**Trial registration:**

Not applicable for prospective cohort studies. Ethics approval prior to study EK 19/2009.

## Background

The International Association for the Study of Pain defines pain as “an unpleasant sensory and emotional experience associated with actual or potential tissue damage or described in terms of such damage” [1]. Neck pain is a common complaint throughout the world and experienced by people of all ages, including children and adolescents [2]. In the Global Burden of Disease 2010 study, neck pain is ranked the fourth leading cause of disability (measured in years lived with disability (YLDs) with an estimated global age-standardised point prevalence of neck pain around 4.9% [3], with about 50% of the patients experiencing persistent pain after 1 year [4]. Even though the age and sex distribution across regions is quite similar, slightly more women (5.8%) than men (4.0%) seem to suffer from neck pain [3]. However, the prevalence estimates of different studies show remarkable heterogeneity [2, 3, 5–9]. These variations are most likely caused by diversity in the case definition (i.e. duration of symptoms, anatomical location), inclusion/exclusion criteria and variations in population [3, 5]. Most studies estimate a 12-month prevalence between 30% to 50% in the adult general population with a prevalence peak in middle age [5, 9–11]. The high incidence of neck pain in the general population and the associated distress make neck pain patients common recipients of medical and chiropractic treatment. In chiropractic practice, neck pain patients are second only to low back patients in their frequency [10, 12, 13].

The disability and economic costs associated with neck pain have a large impact on individuals, their families, healthcare systems and businesses [5, 8, 14, 15]. Calculating the exact health costs is not straightforward. Costs vary depending on the severity of symptoms and the duration of work absence. For a specific calculation, several factors have to be considered and the effective costs are divided into direct costs by detection, treatment, rehabilitation and prevention of the disease and indirect costs caused by disability, absence from work or loss of productivity in an employee while they are at work [8, 14]. With longer duration of symptoms and therefore often associated work absenteeism, indirect costs rise. Borghouts et al. [16] estimated that in 1996, The Netherlands spent 1% of their total health care expenditures on neck pain. Of this only 23% were direct costs, while indirect costs amounted to 77% [16].

As a non-invasive treatment method, chiropractic, including both spinal manipulative therapy (SMT) and mobilization, is suggested as effective therapy for neck pain by recent research [9, 17–19]. Certain medical professionals tend to be concerned about the safety of SMT to the cervical spine, considering a possible damage to the vertebral artery. However, recent research found no evidence of increased risk of vertebral artery injury compared to other primary care physicians [20–22]. Bryans et al. [17] recommend a multimodal approach such as a combination of SMT or mobilization and exercise, massage, patient education etc. for treatment of both acute and chronic neck pain.

Aware of the need for a standardized categorization that could improve prediction of treatment outcome and allow better targeting of care, recent spinal pain research has increasingly been addressing the identification of specific patient subgroups which may have more or less favourable outcomes [23–25]. There have been several studies conducted with the purpose to identify predictors for treatment response of neck pain patients to chiropractic SMT [12, 26–32]. All of these projects conclude the necessity to find more specific definitions and subgroups for neck pain patients, as the large heterogeneity makes comparing different studies a major challenge.

There are various ways to subdivide neck pain patients including gender, age, type of onset, aetiology (mechanical or neuropathic), severity or duration of symptoms. According to The International Association for the Study of Pain, chronic pain is defined as pain which persists past the usual time of healing [1]. Among different ways of categorization, duration of symptoms might even be the strongest predictor of treatment outcome [9]. Various studies found shorter duration of neck pain a predictor of a favourable outcome in neck pain patients [26, 27, 29], while similar studies discussing low back pain also demonstrated the importance of duration and extent of symptoms [33, 34]. However, there is no consistent definition in the literature relating to the time frames used to categorize patients since onset of pain [35]. While most clinical studies agree about the time cut-off point for “chronic” patients at >3 or >6 months [1, 36], categorisation of “acute” neck pain varies widely from <1 week [19], <3 weeks [28], <4 weeks [19, 27, 29], <6 weeks [9] or even longer [23]. However, it is unknown if patients in these various ‘acute’ categories have similar outcomes when receiving similar treatments. Peterson et al. [29] stated that acute neck pain patients (0–4 weeks of symptoms) have higher pain levels and disability before chiropractic treatment but improve faster within the first 3 months compared to chronic patients (> 3 months of symptoms). At the same time, early improvement after initial treatment has been shown to be a strong positive predictive factor for a favourable treatment response also in chronic neck pain patients [29]. Similar results were found investigating predictors of improvement in patients suffering from low back pain [37–39]. However, in acute chiropractic patients treated for low back pain the time frame for categorizing an ‘acute’ onset as 0–4 weeks has recently been challenged [40].

Assuming that the duration of symptoms has a relevant influence on the patient response to the treatment, it is necessary to develop a consistent definition regarding the term “acute” in neck pain research. This may lead to more specific and targeted treatments for certain patients, particularly relating to possible psychosocial factors. To find a more accurate onset of symptoms categorization, the current 0–4 week predefinition of the “acute” subgroup in neck pain patients needs to be investigated.

In a study of low back pain patients receiving chiropractic treatment, differences in outcome within the acute subgroup were reported and the common definition of acute low back pain lasting 0–4 weeks was challenged by suggesting it was too long [40]. The purpose of this study is to explore whether or not the time frame of 0–4 weeks in terms of the definition of “acute” for neck pain patients is determined accurately. Therefore, the objective of this study is to investigate whether or not symptom duration of 0–4 weeks as the definition of ‘acute’ for neck pain patients has the strongest association with outcomes in chiropractic patients compared with other time frames.

## Methods

This is a follow-up study to the prospective cohort study “Predictors of outcome in neck pain patients undergoing chiropractic care: comparison of acute and chronic patients” [29]. It is designed as a prospective cohort observational study with long-term follow-up up to 1 year post treatment. Data was collected between October 2009 and March 2015.

### Patients

Chiropractic practices in Switzerland were asked to contribute patients to this study and 81 of the 260 Swiss chiropractors participated. Selection criteria were patients with age over 18, neck pain of any duration and no chiropractic or manual therapy in the prior 3 months. Patients with contraindications to chiropractic manipulative therapy in the form of specific pathology were excluded. These included acute fractures, tumours, infections, inflammatory arthropathies, Paget’s disease, anti-coagulation therapy, cervical spondylotic myelopathy, known unstable congenital anomalies and severe osteoporosis. For this study, only the data from patients with symptoms between 0 and 12 weeks were used resulting in a sample size of 495 patients. It is unknown what proportion of patients asked to participate in this study by their chiropractors actually agreed.

### Baseline data

For collecting the patient data, notification and instruction about the study and the study protocol were sent to all of the 260 active members of the Association of Swiss Chiropractors. During the annual mandatory postgraduate continuing education convention (CE), verbal instructions outlining the study protocol were given and workshops on the use of outcome measures in clinical practice were conducted by one of the authors. The convention was held promptly prior to the start of data collection with a request to all active members of the association to recruit patients for this study.

Given this was a pragmatic study, no standardized treatment plan or treatment number was given. The chiropractors were especially asked not to change their treatment methods and there were no specific treatments excluded. However, 76% to 100% of the Swiss Chiropractors use “diversified” technique as one of their primary treatments [41]. Other commonly used additional treatments include advice on the activities of daily living, trigger-point therapy, therapeutic exercises, and mobilization techniques [41].

Clinical and demographic baseline data about the patients was provided by the treating chiropractor, including patient age, sex, marital status, paid employment, onset of pain due to trauma or not, the patient’s general health status, associated dizziness, whether or not the patient smokes, current pain medication, duration of complaint, number of previous episodes and if there were signs and symptoms of cervical radiculopathy.

Prior to the initial treatment, each patient was requested by the office staff of the practice to complete a questionnaire assessing their individual impairment, including the numerical rating scale (NRS) for neck pain and a separate NRS for arm pain where 0 = no pain and 10 = the worst pain imaginable. Additionally, patients completed the Bournemouth Questionnaire for neck disability (BQN), which has been translated and validated into the German language [42].

### Outcome measures

For the assessment of outcome, data from the NRS (neck and arm separately), BQN and Patient Global Impression of Change (PGIC) scale [43] were collected. The PGIC is a self-report measure and reports the patient’s perception of the efficacy of treatment. The patient rates his individual impression of overall change on a 7 item scale including the responses “much better”, “better”, “slightly better”, “no change”, “slightly worse”, “worse” and “much worse” [43, 44] (primary outcome measure). The PGIC was dichotomized into ‘improved’ and ‘not improved’ patients. The responses ‘much better’ and ‘better’ were considered ‘improved’ and all other responses ‘not improved’.

One week, 1 month, 3 months, 6 months and 1 year after the initial treatment, the patients were questioned via telephone interviews about their treatment response. The interviews were conducted by trained research assistants blinded to patient or referring chiropractor identity. The time frame when each telephone call should be done was strictly limited (i.e. 6–8 days for 1 week data) [29]. Thus, not every patient could be reached for the telephone interview during the predefined time period but remained in the study if other time periods contained valid data.

### Statistical analysis

According to the duration of symptoms, the patients were divided into three subgroups of high-acute (0–2 weeks of symptoms) (*N* = 274), mid-acute (2–4 weeks of symptoms) (*N* = 62), and subacute (4–12 weeks of symptoms) (*N* = 159). The available statistical power varied depending on the subgroups being compared and the dependent variable being used. For example, a contrast between the 274 high-acute and 159 sub-acute patients would have had 80% power to detect a between group difference in mean neck pain intensity that was as small as 0.6 points on the 0–10 NRS scale, given the standard deviations in the sample and an alpha of 0.06. For all three subgroups, baseline factors were compared using ANOVA for numerical data and the Chi-square test for categorical data. At all follow-up time points the proportions of patients ‘improved’ within each of the 3 groups were compared using the Chi-square test. For the secondary outcomes of NRS change scores (baseline score – follow-up score) and BQN change scores the ANOVA test was used for all follow-up time points as the data was normally distributed. Using the change scores rather than the actual outcome scores usually provides normally distributed data, as in this case, and thus allows the means and standard deviations to be reported.

Additionally, treatment outcomes of high acute (0–2 weeks) vs. midacute (2–4 weeks) as well as midacute vs. subacute (4–12) neck pain patients were compared in order to identify whether the midacute patients behave more similarly to the high acute or the subacute patient groups in their treatment response. The Chi-square test was used for categorical variables (i.e. improvement) and the unpaired Student’s t-test was used for the NRS and BQN change scores.

A *p*-value ≤0.05 was considered statistically significant. For all data analysis SPSS Version 21, IBM, Armonk, New York, USA was used.

### Ethics

Written informed consent was obtained from all patients and ethics approval was obtained from the Canton of Zürich Switzerland ethics committee (EK 19/2009).

## Results

### Baseline characteristics

Of the 260 active members of the Association of Swiss Chiropractors 81 (31%) contributed patients to this study. Baseline data from 495 patients with symptoms of 12 weeks or less were available and included in this study. Of these, 274 were highly acute (0–2 weeks of symptoms), 62 mid-acute (2–4 weeks of symptoms) and 159 subacute (4–12 weeks of symptoms). At the consecutive time points at 1 month, 3 months, 6 months and 1 year after the initial treatment, the numbers of included patients vary. This is a result of the limited time period when each follow-up telephone call was conducted. Some patients could not be reached during the predefined time frame but remained in the study if data from other time points was available.

Baseline characteristics for all three subgroups are shown in Table 1. Significant differences in baseline characteristics within the 3 subgroups are summarised in Table 2. Mid-Acute patients were significantly older than both high-acute as well as subacute patients. No other significant differences between the high-acute and mid-acute subgroups were found. Comparing subacute and mid-acute patients found that subacute patients were less likely to smoke and reported a significantly lower baseline NRS score than the mid-acute subgroup. The subacute patients also had a significantly lower baseline NRS score than high-acute patients, were significantly older than high-acute patients and reported a lower General Health. Additionally, a significantly higher percentage of high-acute patients reported a smoking habit compared to subacute patients.

**Table 1:** Comparison of high-acute, mid-acute and subacute neck pain patients’ baseline pain and disability scores as well as baseline characteristics

	High-acute (0–2 weeks) *n* = 274	Mid-acute (2–4 weeks) *n* = 62	Subacute (4–12 weeks) *n* = 159	*P*-Value
Pre NRSNeck Mean (SD)	6.23 (±2.02)	5.94 (±2.35)	5.41 (±2.30)	0.001*
Pre BQ total Mean (SD)	33.54 (±15.22)	33.94 (±15.25)	31.10 (±16.38)	0.244
Gender	Male: 102 (37.2%) Female: 172 (62.8%)	Male: 27 (43.5%) Female: 35 (56.5%)	Male: 51 (32.1%) Female: 108 (67.9%)	0.255
Age (years) Mean (SD)	38.7 (±12.11)	47.1 (±12.73)	42.8 (±14.54)	0.0001*
General Health	good: 200 (74.9%) average: 55 (20.6%) poor: 12 (4.5%)	good: 38 (62.3%) average: 20 (32.8%) poor: 3 (4.9%)	good: 93 (59.6%) average: 55 (35.3%) poor: 8 (5.1%)	0.013*
Radiculopathy present (yes)	37 (13.7%)	12 (19.4%)	27 (17.4%)	0.399
Trauma onset (yes)	39 (14.2%)	8 (13.1%)	20 (12.7%)	0.893
Smoker (yes)	59 (22.1%)	14 (23.3%)	18 (11.5%)	0.016*

*NRS* numerical rating scale for pain, *BQ* Bournemouth questionnaire, *SD* Standard Deviation. * = *p* ≤ 0.05

**Table 2:** *P*-values obtained when Comparing High-acute vs. Mid-acute, Mid-acute vs. Subacute and High-acute vs. Subacute Patients in terms of the Baseline characteristics significant in Table 1

	High-acute vs. Mid-acute	Mid-acute vs. Subacute	High-Acute vs. Subacute
Pre NRS Neck	0.322	0.124	0.0001*
Age (years)	0.0001*	0.043*	0.002*
General Health	0.115	0.935	0.003*
Smoker (yes)	0.971	0.046*	0.009*

*NRS* numerical rating scale for pain, *BQ* Bournemouth questionnaire; * = *p* ≤ 0.05

### Outcomes

**Fig. 1:**
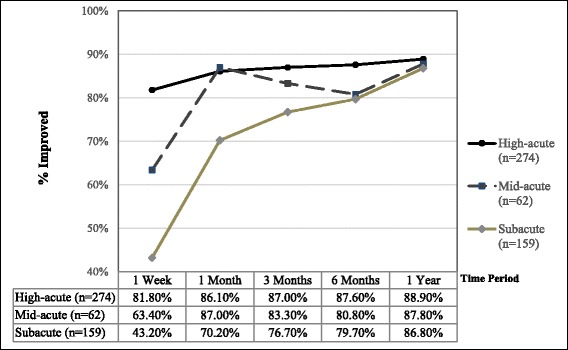
Primary outcome: Patients ‘improved’ at given outcome time points

Improvement on the PGIC scale was the primary outcome measure of the study. The percentage of patients ‘improved’ amongst the three subgroups is shown in Fig. 1. The high-acute subgroup had a significantly higher percentage of ‘improved’ patients compared to the mid-acute patients only at the 1 week time point (*p* = 0.015) (Table 3), whereas the significant differences between high-acute and subacute patients persisted to the 3 month time point (*p* = 0.0001 at 1 week and 1 month; *p* = 0.018 at 3 months) (Table 3 and Fig. 1). Mid-acute patients had significantly better outcomes compared to the subacute patients at both the 1 week (*p* = 0.039) and 1 month (*p* = 0.025) time points. At the 6 month and 1 year time points significant differences between the 3 subgroups were no longer found (Table 3).

**Table 3:** *P*-value Results Comparing the Proportion of Patients Reporting Clinically Relevant ‘Improvement’ for the 3 different Chronicity Categories at all time Points

	High-acute vs. Mid-acute	Mid-acute vs. Subacute	High-Acute vs. Subacute
1 week	0.015*	0.039*	0.0001*
1 month	1.000	0.025*	0.0001*
3 months	0.630	0.420	0.018*
6 months	0.284	1.000	0.068
1 year	1.00	0.565	0.146

* = *p* ≤ 0.05

The secondary outcome data showing the change scores (baseline value – follow-up value) of NRS (neck), and the BQN for all outcome time points are shown in Table 4. Except for the 1 week and 6 months outcome time points (NRS change), the mid-acute patient subgroup did not differ significantly from the high-acute subgroup or the subacute patient subgroup regarding the investigated patient-reported scores. High-acute and subacute patient cohorts however, report significantly different scores at every time point except at 1 week (BQ change).

**Table 4:** Comparison of NRS and BQ Change Scores for Patients in the 3 Chronicity Categories at all Follow-up Time Points

	Number	High-acute (0–2 weeks) Mean (SD)	Number	Mid-acute (2–4 weeks) Mean (SD)	Number	Sub-acute (4–12 weeks) Mean (SD)	*P* Values
Change NRS Neck 1 week	220	3.33 (±2.49)	42	2.52 (±2.23)	124	1.66 (±2.28)	High-acute vs. Mid-acute: *p* = 0.053 Mid-acute vs. Subacute**:** ***p*** = 0.035* High-acute vs. Subacute: ***p*** = 0.0001*
Change BQ total 1 week	221	8.29 (±14.34)	42	7.47 (±13.98)	125	6.75 (±14.09)	High-acute vs. Mid-acute: *p* = 0.733 Mid-acute vs. Subacute: *p* = 0.775 High-acute vs. Subacute: *p* = 0.336
Change NRS Neck 1 month	224	4.26 (±2.64)	54	3.55 (±2.96)	139	2.83 (±2.63)	High-acute vs. Mid-acute: *p* = 0.086 Mid-acute vs. Subacute**:** *p* = 0.098 High-acute vs. Subacute: ***p*** = 0.0001*
Change BQ total 1 month	223	21.87 (±16.99)	54	18.33 (±18.07)	140	15.65 (±17.53)	High-acute vs. Mid-acute: *p* = 0.177 Mid-acute vs. Subacute**:** *p* = 0.344 High-acute vs. Subacute: ***p*** = 0.001*
Change NRS Neck 3 months	223	4.59 (±2.75)	54	4.18 (±2.73)	131	3.30 (±2.92)	High-acute vs. Mid-acute: *p* = 0.324 Mid-acute vs. Subacute**:** *p* = 0.061 High-acute vs. Subacute: ***p*** = 0.0001*
Change BQ total 3 months	221	25.54 (±16.40)	54	24.36 (±17.13)	132	19.14 (±19.17)	High-acute vs. Mid-acute: *p* = 0.637 Mid-acute vs. Subacute**:** *p* = 0.084 High-acute vs. Subacute: ***p*** = 0.001*
Change NRS Neck 6 months	227	4.93 (±2.47)	52	3.95 (±2.56)	132	3.31 (±2.61)	High-acute vs. Mid-acute: ***p*** = 0.012* Mid-acute vs. Subacute**:** *p* = 0.136 High-acute vs. Subacute: ***p*** = 0.0001*
Change BQ total 6 months	192	18.74 (±16.04)	38	17.34 (±14.47)	109	11.54 (±16.58)	High-acute vs. Mid-acute: *p* = 0.618 Mid-acute vs. Subacute**:** *p* = 0.058 High-acute vs. Subacute: ***p*** = 0.0001*
Change NRS Neck 1 year	216	4.91 (±2.60)	49	4.17 (±2.63)	127	3.41 (±2.78)	High-acute vs. Mid-acute: *p* = 0.073 Mid-acute vs. Subacute**:** *p* = 0.101 High-acute vs. Subacute: ***p*** = 0.0001*
Change BQ total 1 year	216	26.41 (±17.03)	49	22.56 (±16.49)	128	19.42 (±16.50)	High-acute vs. Mid-acute: *p* = 0.151 Mid-acute vs. Subacute**:** *p* = 0.260 High-acute vs. Subacute: ***p*** = 0.0001*

*SD* Standard Deviation, *NRS* Numerical rating scale for pain, *BQ* Bournemouth questionnaire; * = *p* < 0.05

## Discussion

The primary outcome measure of ‘clinically relevant improvement’ in this study only showed a significant difference between the high-acute patients (0–2 weeks of symptoms) and mid-acute patients (2–4 weeks of symptoms) at the data collection time point of 1 week with a higher proportion of the high acute patients reporting improvement. There were no further significant differences in the primary outcome for the later data collection time points detected for these two subgroups. Thus this is different from the results obtained for the chiropractic low back pain study where there were more significant differences between the patients in these two ‘acute’ time frames [40].

Comparison of mid-acute and subacute neck pain patients showed some significant differences at the 1 week and 1 month time points, with mid-acute patients still having a significantly better outcome than subacute patients. After 6 months, no further significant differences were found between the three subgroups. Treatment outcomes level off at the 6-month follow-up time point with more than 86% of the patients in all three subgroups considered as ‘improved’. Patients with mid-acute (2–4 weeks) duration of symptoms showed no significant differences either to the high-acute (0–2 weeks) or the subacute (4–12 weeks) subgroups with their proportions of patients responding to chiropractic treatment falling between the high-acute and subacute subgroups.

Analyzing the secondary outcome measures of the BQ and NRS change scores, mid-acute patients also do not differ significantly from either the high-acute subgroup or the subacute patient subgroup whilst high-acute and subacute patient cohorts report significantly different scores at almost every time point.

The results of this study demonstrate a time-dependent converging of outcomes among the three subgroups. Statistically significant differences between the high-acute (0–2 weeks) and mid-acute (2–4) subgroups could only be reported 1 week after start of treatment and thus the clinical relevance of this result is negligible. The findings of this study lead to the conclusion that a categorization of acute pain according to 0–4 weeks of symptoms should be preferred to the suggested 0–2 weeks of symptoms for neck pain patients. In current research, the most often used time frame for acute neck pain uses symptom duration of 0–4 weeks [19].

The findings of this neck pain study are surprisingly different from a parallel study on low back pain patients in similar Swiss chiropractic settings [40]. Investigating low back pain patients receiving chiropractic treatment, Mantel et al. reported significant differences in the outcome of low back pain patients with 0–2 and 2–4 weeks of symptoms at the time points of 1 week, 1 month, and 6 months and those authors stated that for low back pain patients undergoing chiropractic treatment that 0–4 weeks of symptoms as the definition of ‘acute’ low back pain is too long and that a definition of 0–2 weeks is preferable [40].

OECD (Office for Economic Cooperation and Development) guidelines [45] - as well as recommended tools for assessing quality of research [46] request that the study population in a research project should be clearly specified and predefined. From a research perspective, the division of patient groups according to their duration of symptoms depicts a measurable and reproducible way of subgrouping. However, interpreting clinical outcome data of acute neck pain patients bears a major challenge. The entity of neck pain depicts more accurately a symptom than a medical condition. In the absence of acute trauma, neck pain is often a slowly developing condition and the exact time of onset may be difficult to pin point, depending on the pain threshold level for each patient. Thus, determining whether a patient fits into the 0–2 week or 2–4 weeks’ time period is not always as precise as we would like to think. Additionally, the aetiology of neck pain is variable. This study has its focus on the outcome of chiropractic patients. Therefore, the individual cause of neck pain may vary between neuropathic or nociceptive (mechanical, myofascial etc.) pain. Differences in the baseline characteristics of the three subgroups might be explained through the small number of patients particularly in the 2–4 week onset group, and different causes of neck pain being unequally represented in each subgroup. There were no differences in recruiting procedure between the subgroups. Baseline differences were assessed as well and taken into consideration interpreting the clinical outcome. So far, there is very limited evidence regarding the influence of baseline factors on the outcome of neck pain patients [47, 48]. Even though neck pain patients depict a very heterogeneous study population, current literature suggests a predictive value of certain baseline factors [15, 27, 47, 49]. In a 2007 systematic review, Mallen et al. note that baseline pain characteristics (pain intensity, duration, number of previous episodes and multiple-site pain), levels of disability and psychological factors (anxiety, depression, adverse coping strategies, low social support) were all associated with subsequent outcome in musculoskeletal pain [50].

Comparing the three subgroups in this study, baseline differences were registered for baseline severity of pain, mean age, general health and smoking habit. There were no significant differences detected for the other baseline factors including the baseline BQ score, patient gender as well as pain onset due to trauma or pain accompanied with radiculopathy. Several studies report the number of previous episodes as one of the most valuable predictive factors for outcome [27, 30, 47, 49]. However, this was not assessed in this study as that data was previously published [30].

From the 3 subgroups evaluated in this study, both high-acute and mid-acute patients were more likely to smoke than subacute patients. Smoking might be a risk factor for the development or exacerbation of pain. Long-time smoking may sensitize pain receptors, decrease pain tolerance, increase pain perception, and has been shown to contribute to pain persistence [51–57].

Additional differences were found between the high-acute and subacute patients. High-acute patients reported a higher severity of pain on the NRS scale than subacute patients. However, these pain levels were obtained upon presentation for treatment and not at the actual onset of symptoms. The difference in baseline pain severity in the high-acute subgroup is most likely an effect of natural history within the acute pain phase [58].

The last difference in the baseline characteristics comparing these 3 subgroups of neck pain patients found that a significantly higher number of patients reported below average health in the subacute group. This may correspond to the results of other studies which report an association between faster recovery and better general health [6, 59, 60].

It is conceivable that subgrouping according to the duration of symptoms remains an artificial and somehow arbitrary way of categorization amongst a very heterogeneous acute neck pain population. The definition of acute and chronic pain by duration of symptoms is predicated on the assumption that acute pain signals a potential tissue damage, whereas chronic pain results from central as well as peripheral sensitization where pain is sustained after nociceptive inputs have diminished [61, 62].

### Implications for future research

Subgrouping patients by means of symptom duration appears to be an easy and objective way of categorisation. However, duration-based definitions can be difficult to apply in terms of recurrent pain or pain with gradual onset. For multiple reasons, current research criticises pain definition solely by duration, suggesting pain to be a multi-dimensional concept [5, 47, 62, 63]. To embrace the complex and multi-dimensional concept of neck pain, we recommend for future research the use of either multidimensional or multiple categorisation criteria additional to the duration of symptoms as suggested by The Bone and Joint Decade 2000–2010 Task Force on Neck Pain and Its Associated Disorders [64].

### Limitations

There are limitations to this study. This is not a randomized clinical trial but a prospective cohort observational study. As there is no control group in this study, outcomes of the patients cannot be definitely attributed to the treatment but may correspond with the natural course of healing process within the acute pain phase [58].

Baseline information for this study was collected using paper questionnaires. However, follow-up data was collected via telephone interviews. Multiple studies have detected a false positive effect on outcomes with patients being more likely to report more favourable outcome to the interviewer [63, 65, 66]. Even though the telephone interviews were conducted by anonymous research assistants at the university, unknown to the patients, these effects cannot be excluded.

An important limitation to this study is the smaller sample size in the mid-acute patient group. Additionally, not every patient could be reached at the different follow up time points, especially at the follow up time point 1 week post treatment resulting in there being fewer patients because of the narrow time window allowed. As this was a secondary analysis on data previously collected for another study, power calculations to determine an adequate sample size for the 2–4 week subgroup were not done prior to data analysis as no additional patients could be included.

It is also not known what proportion of patients asked to participate in this study by their treating chiropractor actually agreed to participate.

Although the use of the Bournemouth questionnaire may appear to be a limitation in this study due to the fact it only contains 7 subscales, previous studies have found it to be a reliable and valid instrument and more responsive to change compared to the Neck Disability Index and the Neck Pain and Disability Scale [42, 67].

## Conclusion

The time period with 0–4 weeks of symptoms as the definition of “acute” neck pain should be maintained. Patients with a shorter period (0–2 weeks) of symptoms recover faster than patients with a longer period of symptoms (2–4 weeks) but this difference is only statistically significant at the 1 week and 1 month time periods. These results for neck pain patients are different from those obtained in the similar study investigating acute low back pain patients where the 0–2 weeks time period as the definition of ‘acute’ was recommended. Medium-term and long-term outcome is favourable for acute as well as subacute neck pain patients, independent of the exact duration of symptoms.

## Abbreviations

BQ: Bournemouth questionnaire
BQN: Bournemouth questionnaire for neck pain
NDI: Neck disability index
NPAD: Neck pain and disability scale
NRS: Numerical pain rating scale
OECD: Organization for Economic Cooperation and Development
PGIC: Patient’s Global Impression of Change Scale
